# Humoral immunity to memory antigens and pathogens is maintained in patients with chronic kidney disease

**DOI:** 10.1371/journal.pone.0195730

**Published:** 2018-04-16

**Authors:** Nadezhda A. Wall, C. Coral Dominguez-Medina, Sian E. Faustini, Charlotte N. Cook, Andrew McClean, Mark D. Jesky, Marisol Perez-Toledo, Matthew D. Morgan, Alexandra G. Richter, Charles J. Ferro, Paul Cockwell, Paul A. Moss, Ian R. Henderson, Lorraine Harper, Adam F. Cunningham

**Affiliations:** 1 University of Birmingham, Birmingham, United Kingdom; 2 University Hospitals of North Midlands NHS Trust, Stoke-on-Trent, United Kingdom; 3 University Hospitals Birmingham NHS Foundation Trust, Birmingham, United Kingdom; Icahn School of Medicine at Mount Sinai, UNITED STATES

## Abstract

Patients with chronic kidney disease (CKD) have an increased risk of infection and poorer responses to vaccination. This suggests that CKD patients have an impaired responsiveness to all antigens, even those first encountered before CKD onset. To examine this we evaluated antibody responses against two childhood vaccine antigens, tetanus (TT) and diphtheria toxoids (DT) and two common pathogens, cytomegalovirus (CMV) and *Salmonella enterica* serovar Enteritidis (SEn) in two independent cohorts consisting of age-matched individuals with and without CKD. Sera were evaluated for antigen-specific IgG titres and the functionality of antibody to SEn was assessed in a serum bactericidal assay. Surprisingly, patients with CKD and control subjects had comparable levels of IgG against TT and DT, suggesting preserved humoral memory responses to antigens encountered early in life. Lipopolysaccharide-specific IgG titres and serum bactericidal activity in patients with CKD were also not inferior to controls. CMV-specific IgG titres in seropositive CKD patients were similar or even increased compared to controls. Therefore, whilst responses to new vaccines in CKD are typically lower than expected, antibody responses to antigens commonly encountered prior to CKD onset are not. The immunodeficiency of CKD is likely characterised by failure to respond to new antigenic challenges and efforts to improve patient outcomes should be focussed here.

## Introduction

Chronic kidney disease (CKD) is an important global clinical problem and is defined and staged by measures of kidney function, the estimated glomerular filtration rate (eGFR), and kidney damage, primarily albuminuria. [1] Significant renal impairment (eGFR<60ml/min/1.73m^2^, CKD stages G3-5) is found in approximately 6% of the UK population and its prevalence increases markedly with advancing age, affecting more than 30% of individuals aged 75 years and over. [2]

Reduction in eGFR or increase in albuminuria are both independently associated with all-cause mortality and progression to severe CKD–end stage renal disease (ESRD), which may require renal replacement therapy (RRT). [3, 4] ESRD is associated with a marked increased risk of infection and its resultant morbidity and mortality. [5] Indeed, one year mortality in patients with ESRD is typically greater than 10%, with infection accounting for almost 1 in 5 deaths, [6] and mortality related to sepsis is 30–50 times greater than the general population. [7] A graded association with the incidence of infection and resultant hospitalisation and mortality has also more recently been described in less severe CKD. [8, 9]

Amongst the reasons for the observed increased susceptibility to infection is that public health measures to reduce infection, such as vaccination, are not as effective in CKD as in the general population. Studies have consistently shown that patients with ESRD exhibit a lower seroconversion rate, lower peak antibody titre and faster decline in protective antibody titres than healthy subjects. [10–12] Reflecting what is observed for the risk of infection, there is also a graded reduction in vaccine responses with worsening renal impairment in less severe CKD, and vaccination is currently recommended early in the disease to maximise humoral response. [13–15]

Collectively, these observations have led to a dogma that patients with CKD are immunodeficient and have a defective capacity to mount and maintain effective responses to antigens as compared to the general population. Host immune function has mainly been studied in the ESRD population and findings typically include lymphopenia and contraction of naïve T and B lymphocyte pools. [16] Lower numbers of circulating memory B cells have also been reported in children with severe CKD, [17] together with increased B cell apoptosis in both adults and children with ESRD. [18, 19] These features would suggest that, in addition to reduced responses to newly encountered antigens, there may also be a reduction in immune memory to previously encountered antigens as a result of increased B cell death. Immune function in less severe CKD has not yet been comprehensively characterised, but, as renal disease is a continuum, it is reasonable to suppose that alterations in immune function seen in ESRD start early in the course of CKD, just as other metabolic abnormalities associated with renal disease develop long before severe disease is established.

The effect of less severe CKD on already established immunity to previously encountered antigens is not well explored. Our understanding in this area could be enhanced by assessing the level and functional activity of antibodies against commonly encountered pathogens and vaccines. In this study we have assessed levels of IgG against two vaccine antigens (tetanus toxoid (TT) and diphtheria toxoid (DT)), the bacterium *Salmonella enterica* serovar Enteritidis (SEn) and the viral pathogen cytomegalovirus (CMV), in two independent cohorts of patients with moderate/severe CKD not requiring RRT and age-matched controls. These antigens were chosen as epidemiological evidence suggests that they are all typically encountered by early adulthood. [20–23] Unexpectedly, antibody responses to the different antigens were at least equivalent in patients with CKD compared to healthy controls. This indicates that humoral responses can be maintained to some antigens in patients with CKD and thus the disease does not necessarily induce a global immunodeficiency, but one that reflects when the antigen was encountered.

## Results

### Study population

43 patients with CKD and 39 age-matched controls were initially investigated (hereafter referred to as cohort 1) and their demographics and laboratory data are presented in Table 1. Patients with CKD from this cohort had a median eGFR of 25ml/min/1.73m^2^ (with majority of patients classified as CKD stage G4) and controls 81.5ml/min/1.73m^2^. The most common identified primary renal diagnoses were ischaemic/hypertensive nephropathy in 23%, polycystic kidney disease in 21%, diabetic nephropathy in 19%. Patients with CKD had a minimum of 12 months’ specialist nephrology follow-up at time of study entry. Just over one third of cohort 1 CKD patients had a diagnosis of diabetes mellitus (DM)– 35%, compared with none of the control subjects.

**Table 1:** Summary of demographic, clinical and laboratory data for cohort 1 patients with CKD and controls.

	Controls (n = 39)	CKD (n = 43)	P value
**Age, years**	57 (15)	63 (28)	0.10^a^
**Male: n (%)**	22 (57)	12 (28)	0.01^b^
**eGFR, ml/min/1.73m**^**2**^	82 (16)	25 (13)	<0.0001^a^
**CKD stage G3: n (%)**	-	16 (37)	
**CKD stage G4: n (%)**	-	23 (54)	
**CKD stage G5: n (%)**		4 (9)	
**Cause of CKD: n (%)**			
**Hypertensive / ischaemic nephropathy**	-	10 (23)	
**Diabetic nephropathy**	-	8 (19)	
**IgA nephropathy**	-	5 (12)	
**Polycystic kidney disease**	-	9 (21)	
**Obstructive uropathy**	-	1 (2)	
**Other / uncertain**	-	10 (23)	
**Diabetes mellitus: n (%)**	0	15 (35)	<0.0001^b^
**hsCRP, mg/l**	0.90 (2.19)	2.10 (6.85)	<0.01^a^
**WCC (x10**^**9**^**/l)**	5.6 (1.7)	7.5 (3.1)	<0.0001^a^
**Neutrophil count (x10**^**9**^**/l)**	3.3 (1.4)	4.7 (2.0)	<0.0001^a^
**Lymphocyte count (x10**^**9**^**/l)**	-	1.6 (0.6)	-

Medians and interquartile range shown unless indicated.

^a^2-tailed Mann-Whitney test;

^b^2-tailed Fisher’s exact test; significance is defined as p<0.05. eGFR–estimated glomerular filtration rate, hsCRP–highly sensitive C-reactive protein, WCC–white cell count.

In order to confirm our findings, we then evaluated a second independent cohort of 25 patients with CKD and 20 age-matched healthy controls (hereafter referred to as cohort 2) and their demographics and laboratory data are presented in Table 2.

**Table 2:** Summary of demographic, clinical and laboratory data for cohort 2 patients with CKD and controls.

	Controls (n = 20)	CKD (n = 25)	P value
**Age, years**	72 (9)	73 (9)	0.46^a^
**Male: n (%)**	9 (45)	17 (68)	0.14^b^
**eGFR, ml/min/1.73m**^**2**^	75 (16)	23 (13)	<0.0001^a^
**CKD stage G3: n (%)**	-	7 (28)	
**CKD stage G4: n (%)**	-	18 (72)	
**CKD stage G5: n (%)**		0	
**Cause of CKD: n (%)**			
**Hypertensive / ischaemic nephropathy**	-	10 (40)	
**Diabetic nephropathy**	-	2 (8)	
**IgA nephropathy**	-	0	
**Polycystic kidney disease**	-	0	
**Obstructive uropathy**	-	0	
**Other / uncertain**	-	10 (40)	
**Diabetes mellitus: n (%)**	1 (5)	18 (72)	<0.0001^b^
**hsCRP, mg/l**	0.84 (1.11)	5.58 (6.11)	<0.0001^a^
**WCC (x10**^**9**^**/l)**	6.0 (2.7)	7.4 (3.2)	<0.01^a^
**Neutrophil count (x10**^**9**^**/l)**	3.4 (1.8)	5.0 (3.4)	<0.001^a^
**Lymphocyte count (x10**^**9**^**/l)**	1.7 (0.78)	1.3 (1.0)	0.10^a^

Medians and interquartile range shown unless indicated.

^a^2-tailed Mann-Whitney test;

^b^2-tailed Fisher’s exact test; significance is defined as p<0.05. eGFR–estimated glomerular filtration rate, hsCRP–highly sensitive C-reactive protein, WCC–white cell count.

Individuals from cohort 2 had similar levels of kidney function (eGFR) as cohort 1, but both the control and CKD arms were significantly older in cohort 2 (Mann Whitney 2-tailed p<0.01). Cohort 2 patients with CKD had a median of 2.5 years follow-up by specialist nephrology services (range 0.5 to 16 years) and the most common identified primary renal diagnosis was hypertensive/ischaemic nephropathy (40%), although a large number of individuals (40%) had uncertain CKD aetiology in the context of multiple comorbidities including diabetes, hypertension and cardiovascular disease. There was a significantly higher proportion of individuals with diabetes in cohort 2 CKD patients than in cohort 1 (72% versus 35%, Mann Whitney 2-tailed p<0.01). None of the individuals in cohorts 1 or 2 had any known active malignancy, autoimmune/inflammatory disease (including immune-mediated renal disease) and were not receiving immunosuppressive therapies at the time of entry to the study. Although there was no known active chronic inflammatory disease in CKD patients from either cohort, these patients had significantly higher markers of inflammation than controls, including total white cell count (WCC), neutrophil count and serum high sensitivity C-reactive protein (hsCRP).

### Patients with CKD have similar IgG levels to historical antigens as healthy individuals

Humoral responses to historical antigens in patients with CKD and healthy controls were evaluated by measuring serum anti-TT and anti-DT IgG using a Luminex assay. [24] None of the study subjects had been given any vaccinations as part of this investigation, so measuring anti-TT and DT IgG titres in this population reflects maintenance of a response from previous exposure. It is currently not recommended in the UK to routinely revaccinate adult patients with CKD with either TT or DT more frequently than the general population [22, 23], so it is unlikely that individuals with CKD have had more immunizations or more recent immunizations than controls. Therefore, it is reasonable to conclude that persistent IgG to TT and DT reflect long-term immune responses to these two vaccine antigens.

**Fig 1:**
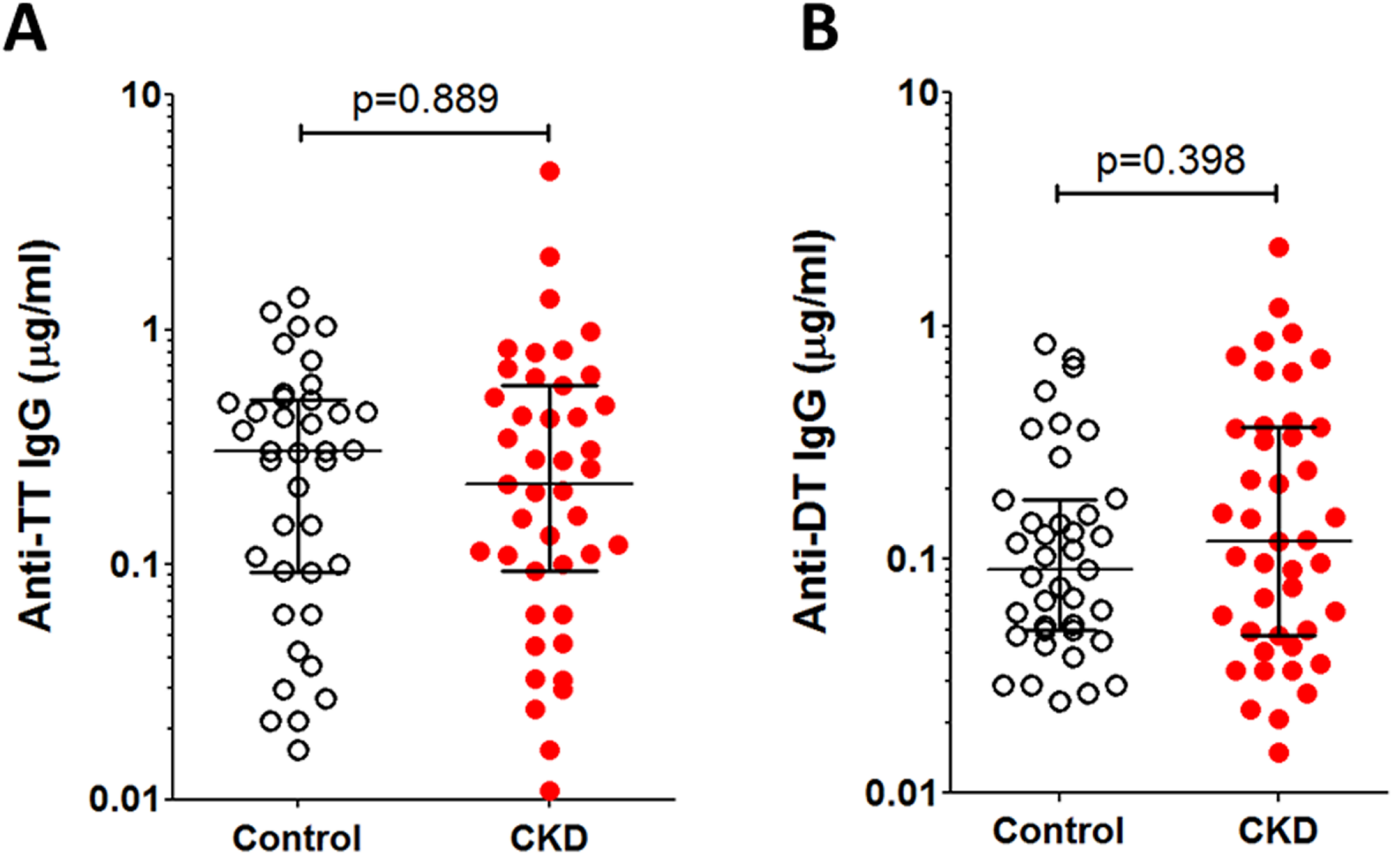
Anti-TT and anti-DT IgG titres in cohort 1 patients with CKD and controls. Antigen-specific IgG titres (μg/ml) in cohort 1 CKD patients and age-matched controls (n = 43 and 39 respectively) are shown with medians and interquartile range. A–anti-TT IgG; B–anti-DT IgG. Two-tailed Mann-Whitney p values shown.

No significant differences were observed in anti-TT or anti-DT IgG titres between healthy controls and patients with CKD in cohort 1 (Fig 1). Here, 32 patients with CKD (74%) had levels of anti-TT IgG above the WHO-defined threshold associated with tetanus protection (0.1μg/ml) [25]–hereafter referred to as “protective titre” for brevity, compared to 28 healthy controls (72%). The number of individuals with a protective titre for anti-DT IgG (greater than 0.1μg/ml [26]) was 23 (54%) for patients with CKD and 19 (49%) for healthy controls. No significant difference was observed in the proportions of protected individuals across the two groups for these antigens (Fisher’s exact 2 tailed p = 0.81 and 0.83 for anti-TT and DT IgG respectively). There were no significant correlations between anti-TT or anti-DT IgG and eGFR (Spearman’s rank 2-tailed p = 0.87 and 0.32 respectively) or albumin/creatinine ratio (ACR) (Spearman’s rank 2-tailed p = 0.11 and 0.65 respectively) in the cohort 1 CKD patients. Individual vaccination records were not available for individuals in cohort 1, but it was presumed that all subjects had received tetanus and diphtheria vaccination by early adulthood as routine childhood vaccination was introduced in the UK in 1961 and 1940 for tetanus and diphtheria respectively [22, 23] and catch-up vaccination programmes in older children and adults were subsequently instigated. There was no significant correlation between age and TT or DT IgG titre in either controls or patients with CKD in cohort 1 (Spearman’s rank 2-tailed p = 0.22 and p = 0.20 for patients with CKD, p = 0.54 and p = 0.18 for controls respectively), further supporting our assumption. We did not have data for place of birth for cohort 1 controls (95% of whom were of White British ethnicity), but when TT and DT IgG titres were compared between cohort 1 patients with CKD born in the UK (n = 37) and those born outside the UK (n = 6) no significant differences were seen (Mann-Whitney 2-tailed p = 0.86 and 0.88 respectively).

**Fig 2:**
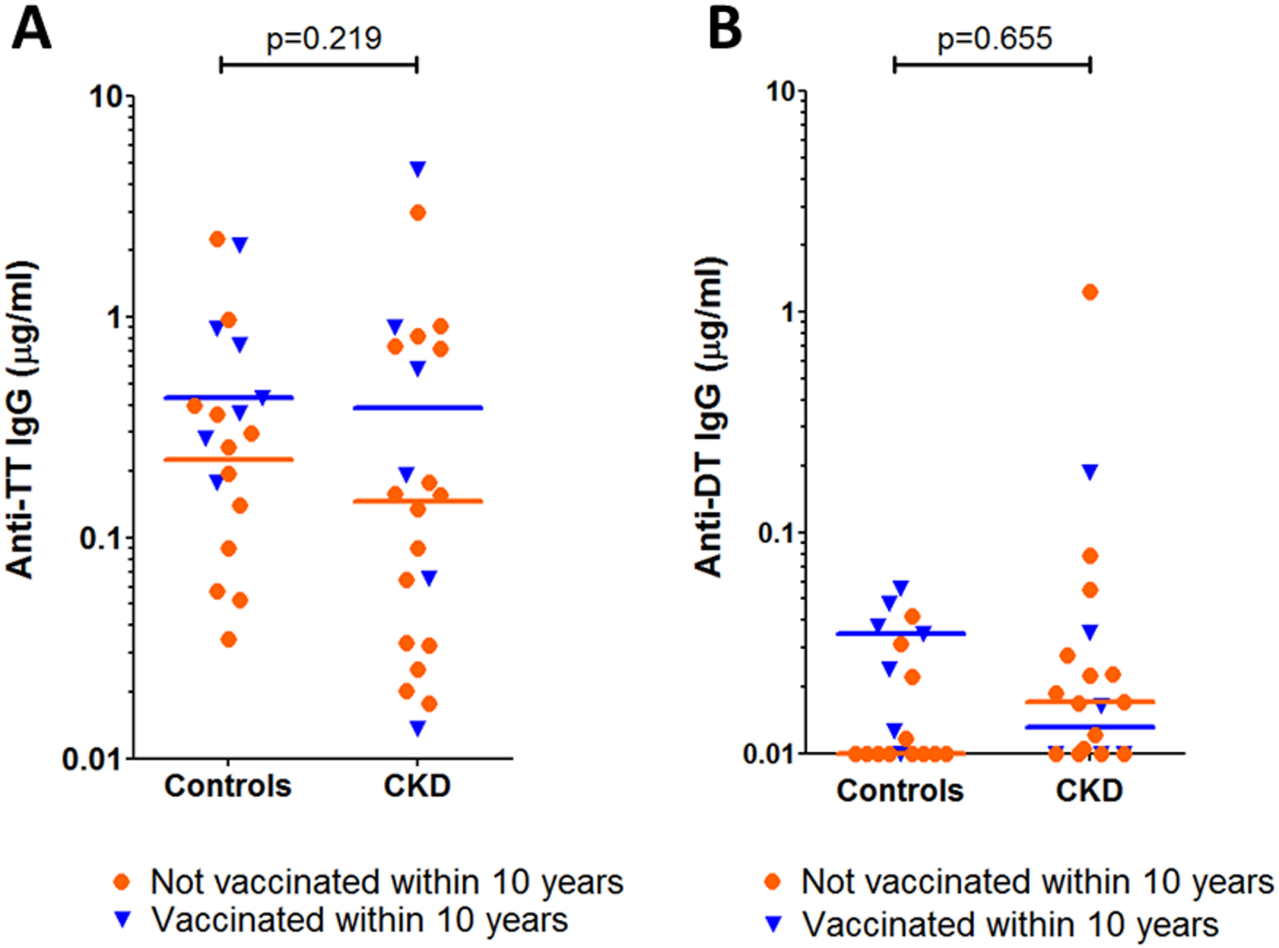
Anti-TT and anti-DT IgG titres in cohort 2 patients with CKD and controls with known vaccination history. Antigen-specific IgG titres (μg/ml) in cohort 2 CKD patients and age-matched controls (n = 22 and 19 respectively) are shown with medians for individuals vaccinated with TT/DT booster in preceding 10 years (blue) and those who were not (orange). The median for anti-DT IgG in controls not boosted within preceding 10 years is 0.01. A–anti-TT IgG; B–anti-DT IgG. Two-tailed Mann-Whitney p values are shown.

To confirm non-inferiority of long-term vaccine-specific antibody responses in patients with CKD, we then evaluated anti-TT and DT IgG levels in patients with CKD and age-matched controls from cohort 2 for whom the vaccination history was known (n = 22 and 19 respectively). There was no significant difference in the numbers of individuals with protective titres of anti-TT and anti-DT IgG between patients with CKD and controls (13 (59%) versus 15 (79%) and 2 (9%) versus 0, respectively) or their absolute anti-TT and anti-DT IgG titres (Fig 2). This was not affected by whether individuals were vaccinated within the preceding decade or not (vaccinated <10 years ago: controls (n = 6) v patients with CKD (n = 7), 2-tailed Mann-Whitney p = 0.73 for TT and p = 0.39 for DT; vaccinated >10 years ago: controls (n = 11) v patients with CKD (n = 16), 2-tailed Mann-Whitney p = 0.40 for TT and p = 0.10 for DT) or if individuals from cohort 2 with indeterminate vaccination history were included in the analysis (3 patients with CKD and 1 control, data not shown). There was no significant difference in TT/DT vaccine coverage in cohort 2 patients with CKD and controls (Fisher’s exact 2-tailed p = 0.737).

### IgG responses to CMV are not inferior in patients with CKD

Sera from study subjects were evaluated for CMV-specific IgG by an enzyme-linked immunosorbent assay (ELISA). This in-house assay was altered between testing sera from cohort 1 and those from cohort 2, therefore the absolute CMV-specific IgG titres are not directly comparable.

The proportion of individuals seropositive for CMV (defined as a result greater than 10 arbitrary units) was not significantly different between CKD and control individuals in either study cohort (cohort 1: 29 patients with CKD (67%) and 22 controls (56%); 2-tailed Fisher’s exact p = 0.37; cohort 2: 21 patients with CKD (84%) and 13 controls (65%), Fisher’s exact 2-tailed p = 0.18). There were no significant differences in rates of CMV seropositivity in either patients with CKD or controls when the two study cohorts were compared (cohort 1 versus cohort 2).

**Fig 3:**
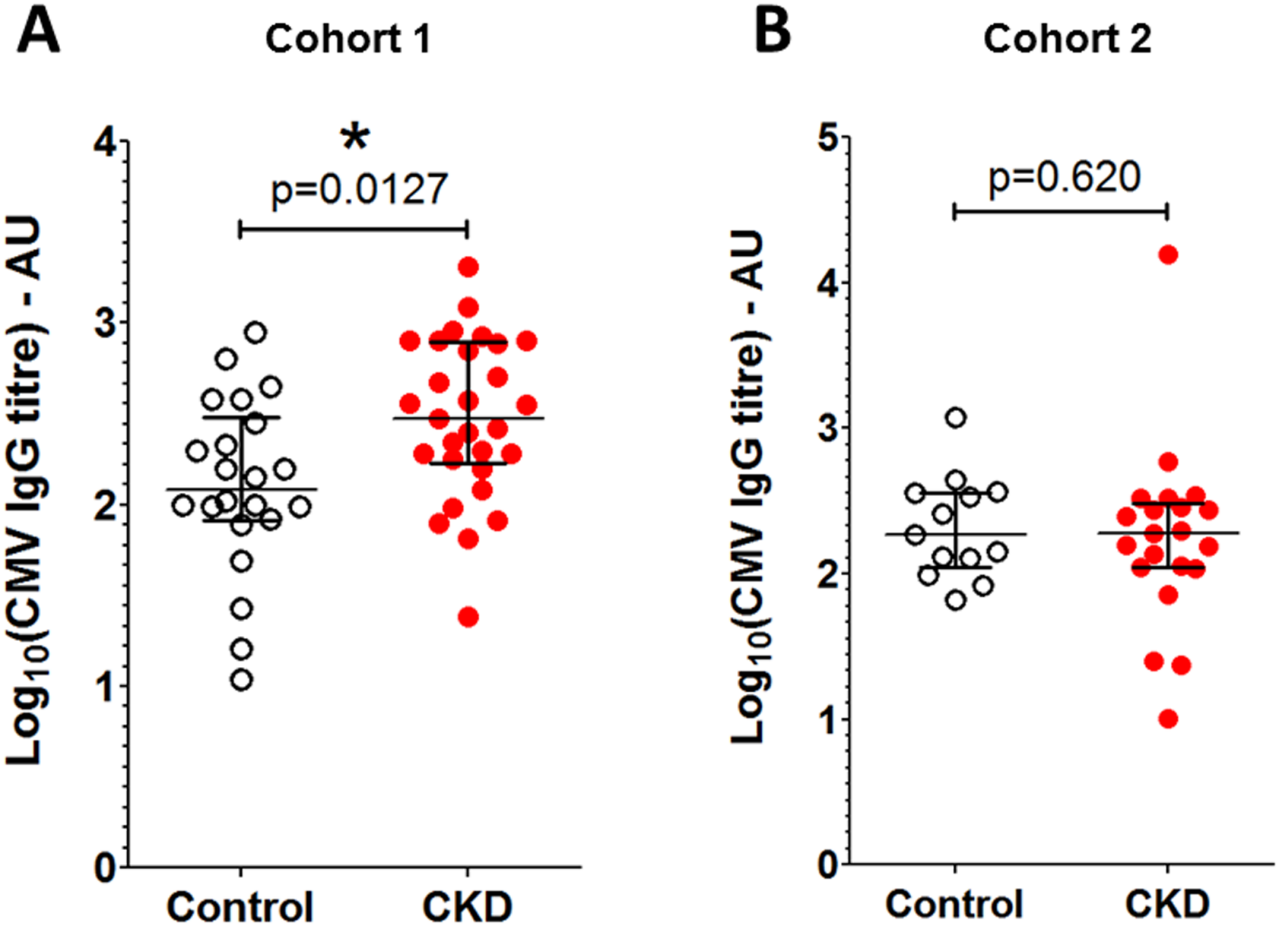
CMV-specific IgG titres in seropositive patients with CKD and controls. Serum CMV-specific IgG titres are shown only for seropositive patients with CKD and controls (ELISA titre greater than 10 arbitrary units, log_10_ = 1) A: cohort 1 (n = 29 patients with CKD and 22 controls). B: cohort 2 (n = 21 patients with CKD and 13 controls). Error bars represent the median and interquartile range. Mann-Whitney 2-tailed p values shown.

When cohort 1 CMV seropositive individuals were evaluated separately, patients with CKD were found to have significantly elevated IgG titres compared to controls (Fig 3A), independent of age (p = 0.02 in a multivariate linear regression model that included age and CKD status). When CMV seropositive individuals only were compared in cohort 2, there were no significant differences seen in the CMV-specific IgG (Fig 3B).

There were no significant correlations between CMV IgG titre and eGFR or ACR in seropositive patients with CKD from either cohort 1 (Spearman’s rank 2-tailed p = 0.36 and 0.10 respectively) or cohort 2 (p = 0.76 and 0.26 respectively). These findings suggest that renal impairment is not associated with a lower anti-CMV IgG response.

### Patients with CKD maintain normal anti-SEn LPS IgG and serum-based bacterial killing

**Fig 4:**
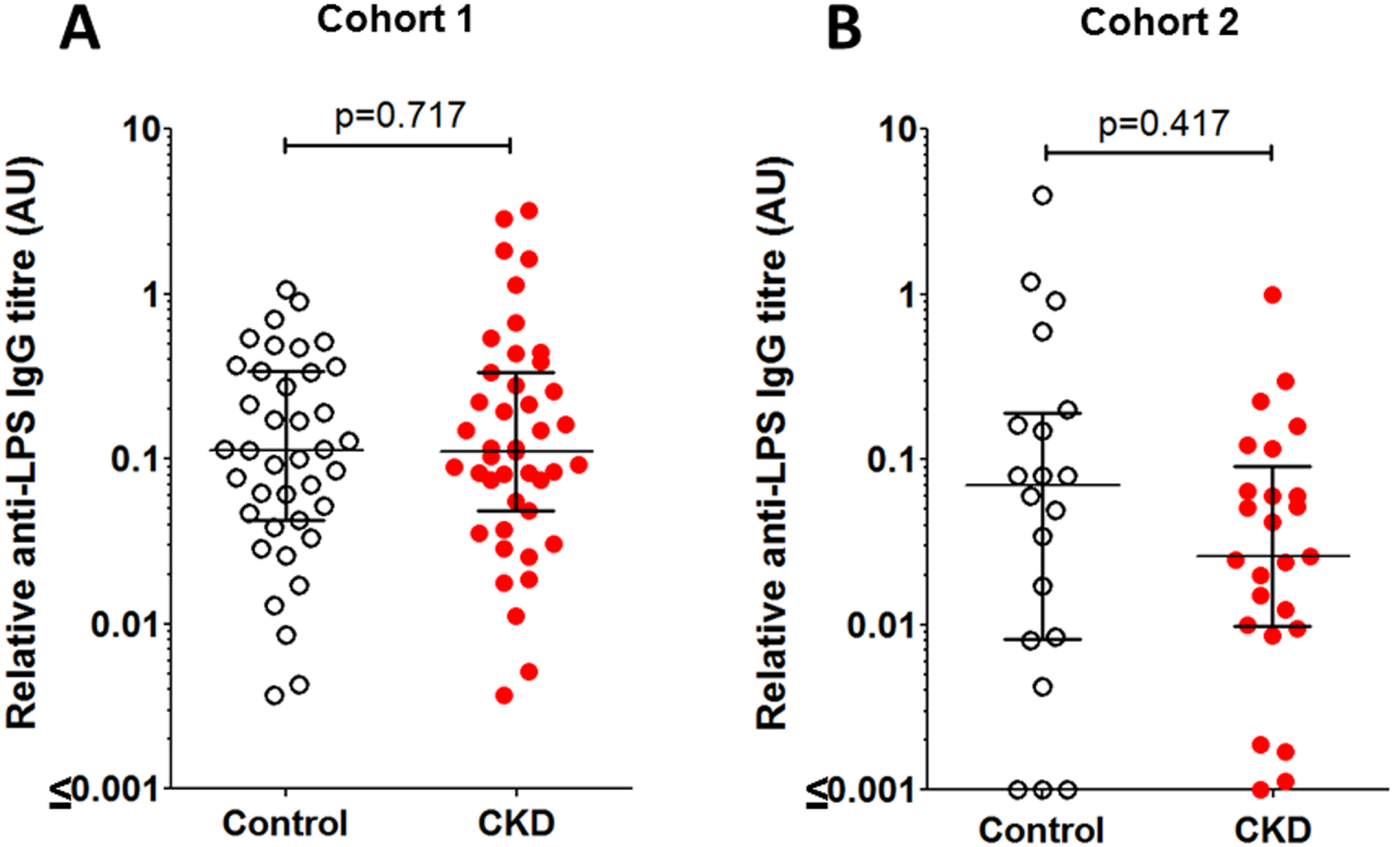
Anti-LPS IgG titres in patients with CKD and controls. Serum anti-LPS IgG titres are represented relative to internal control serum from a single young healthy donor (arbitrary units, AU). A–cohort 1, B–cohort 2. Error bars represent the median and interquartile range. Mann-Whitney 2-tailed p values shown.

Sera from all subjects were tested for anti-SEn LPS IgG by ELISA. Relative anti-LPS IgG titres were not significantly different between patients with CKD and healthy controls in either cohort 1 or 2 (Fig 4).

**Fig 5:**
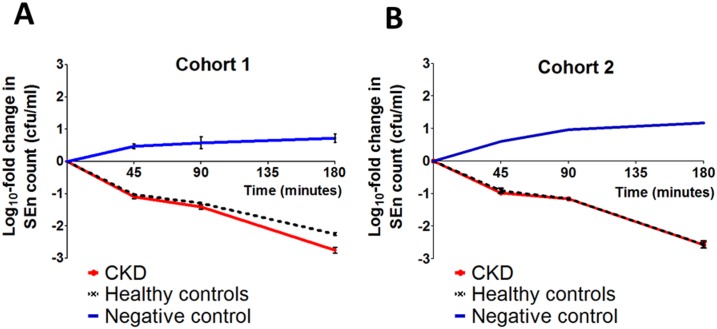
Serum-dependent killing of SEn in patients with CKD and controls. Killing curves of SEn strain D24954 by sera from A—a sub-group of cohort 1 with highest anti-LPS IgG titres: 10 patients with CKD and 9 controls; B–all patients with CKD and controls from cohort 2 (n = 25 and 20 respectively). Negative values correspond to a reduction in SEn compared with starting concentration. Antibody-depleted young healthy control serum (used as standard) served as a negative control. Error bars represent the mean +/- standard error of the mean.

We then went on to investigate the relative functional activity of the antibody and its capacity to activate complement and kill bacteria. Initially, cohort 1 control subjects and patients with CKD with the highest titres of anti-SEn LPS IgG (n = 9 and 10 respectively) were selected for testing in a serum bactericidal assay (SBA). This assay requires both functional antibody and complement for bacterial killing. [27] All sera achieved a greater than 1.5 log_10_ kill of SEn at 180 minutes (Fig 5A), which equates to killing of over 97% of bacteria in the assay. Time-dependent serum killing was not inferior in this subgroup of patients with CKD compared to controls, but actually significantly greater (repeated measures ANOVA p = 0.003). Assay control serum depleted of SEn-specific antibody (negative control) failed to demonstrate any bacterial killing. We repeated the SBA for all patients with CKD and controls in cohort 2, regardless of serum anti-SEn LPS IgG titre (n = 25 and 20, respectively) and found comparable results (Fig 5B). All CKD sera achieved a greater than 1.5 log_10_ kill of SEn at 180 minutes and time-dependent serum killing was not inferior to controls. There was also no correlation between eGFR and degree of bacterial killing in patients with CKD from either cohort 1 or 2. Thus, patients with CKD have similar levels of anti-LPS IgG compared to healthy controls and maintain the capacity to promote complement-dependent, cell-independent killing of bacteria such as *Salmonella*.

## Discussion

This study evaluates serological responses to several historical and persistent antigens in individuals with CKD not requiring RRT. Whilst many reports have evaluated humoral responses to newly encountered antigens, such as vaccines, the effect of CKD on already established humoral memory to previously encountered antigens is not well understood.

After vaccination against hepatitis B, seasonal influenza and pneumococcus, patients with severe CKD consistently show reduced peak antibody titres and poor response longevity when compared to healthy controls. [10, 12, 28] We show, in two independent cohorts consisting of age-matched healthy controls and individuals with CKD, that IgG responses to two common historical vaccine antigens (TT and DT) are comparable in patients with CKD to that of healthy controls, both in terms of absolute values and levels deemed to confer protection against disease. Our findings suggest that the immune impairment seen in CKD is not universal to all antigens encountered across the life course of the individual, but may affect responses to antigens encountered more recently, when disease is established, rather than responses to antigens encountered earlier in life. Furthermore, as most individuals in cohort 2 had not been re-vaccinated within the ten years prior to the study, recent antigen exposure through immunization is unlikely to confound these findings. This is consistent with the concept that patients with CKD have an intact immune system prior to the onset of CKD and are able to mount an adequate humoral response to vaccine antigens encountered early in life. However, what is most interesting is that they can maintain the humoral response to these vaccine antigens in the face of worsening renal function. It has previously been estimated that the half-life of anti-TT and DT responses is approximately 11 and 19 years respectively. [29] Due to the insidious nature of most non-immune pathologies leading to CKD, we can surmise that the relatively short duration of specialist follow-up in our CKD patient cohort is likely to grossly under-estimate the true duration of their renal impairment. It is, therefore, possible that an effect of CKD on long-lived plasma cells would show itself as a change in vaccine-specific IgG titres in our study.

Previous studies evaluating long-term B cell depletion therapy with Rituximab, an anti-CD20 monoclonal antibody, have shown no significant effects on vaccine-specific IgG titres, but a marked reduction in auto-immune antibody titres. [30, 31] Thus, it is possible that the nature of plasma cells and their survival (or CD20 expression) is variable within patients depending on their health status (i.e. autoimmunity, CKD) and age at the time of antigen encounter. This highlights that our understanding of the full mechanisms underlying the maintenance of anti-self and anti-vaccine responses is incomplete.

Our findings of comparable anti-TT and DT IgG titres in 2 independent cohorts of patients with CKD and their age-matched controls is consistent with renal impairment not adversely affecting well-established, long-lived plasma cell populations for antigens first encountered prior to the onset of CKD. This suggests that it is primarily the generation of immune responses to new antigens that is impaired. In support of this idea are the findings of a previous study involving subjects with ESRD that showed faster waning of anti-hepatitis B IgG following vaccination after ESRD was established as compared to historic natural infection [32].

In addition to evaluating responses to protein vaccine antigens, we also examined responses to two pathogens, CMV and SEn. CMV, a betaherpesvirus, establishes life-long persistent infection in humans after primary infection, which often occurs in childhood, and reactivates periodically in response to stressors such as inflammation, infection or immunosuppression. [20, 33, 34] CMV IgG seropositivity is used as a marker of prior infection with a prevalence that increases with age. [20] CMV infection drives the expansion of large numbers of CMV-specific T lymphocytes and this serves to expand the effector memory pool and simultaneously lead to contraction of the naïve T cell pool in older adults. [35, 36] Overall, we observed a non-inferior CMV-specific IgG titre in CKD patients as compared to healthy controls. There was a significantly elevated titre in cohort 1 patients with CKD, independent of age. Interestingly, a similar increase in CMV-specific titres is observed with normal ageing, [37] possibly reflecting higher virus burden in older people [38], and this may account for the findings in cohort 2, who were significantly older than cohort 1. However, the lack of difference detected in cohort 2 may also reflect technical differences in the assay performed. An increase in the CMV-specific T cell pool has been reported in dialysis patients [39, 40] and the magnitude of the humoral and cellular immune responses to CMV have been shown to be closely correlated. [36] CMV infection and high levels of CMV-specific IgG have both been associated with increased risk of mortality [41] and vascular disease [42] in older people and it is noteworthy that a similar association has been observed in patients requiring RRT. [43, 44] As such, our data suggests that CMV burden may be increased in patients with CKD, for example through a relatively increased frequency or magnitude of episodes of CMV reactivation, leading to an enhanced CMV-specific IgG immune response. We therefore feel that the clinical importance of CMV infection and subclinical viral reactivation in the early stages of CKD deserves further investigation.

The immunodominant antigen targeted by IgG after natural infection with SEn is the O-antigen of LPS. Virtually all adults in developed countries develop IgG antibodies to LPS from multiple *Salmonella* serovars by the time of adulthood [21] and in regions such as sub-Saharan Africa this is already seen in children by the age of 4 years. [45] As such, assessment of anti-*Salmonella* LPS antibodies provides a valuable measure of the immune response to a bacterial pathogen that is probably encountered repeatedly throughout life. [21] Unlike some patients with conditions such as HIV or bronchiectasis who can have supra-physiological levels of IgG to LPS that are inhibitory to bacterial killing *in vitro*, [46, 47] all patients with CKD had sera that could kill SEn and anti-LPS IgG titres were similar between patients with CKD and controls. The SBA allows us to investigate cell-free killing of *Salmonella* and depends upon the presence of both intact complement and specific antibodies, which together combine to generate membrane attack complexes resulting in bacterial lysis. Therefore, we can conclude that patients with CKD are able to produce functional pathogen-specific IgG and have intact classical complement activation.

Our study has several strengths including examination of humoral responses in two independent cohorts of age-matched individuals and the exclusion of dialysis therapy as a confounding factor, which is known to independently modulate immune function. [48, 49] Limitations of our study include not having functional antibody data for all the antigens examined and the lack of investigation of antigen-specific T/B cells. Whilst we have focused on the serological response as a proxy for T and B cell responses, deeper insights may be possible if the plasma cell beds in the bone marrow were assessed. Nevertheless, such studies are difficult to undertake for both technical and ethical reasons.

In summary, our results indicate that the secondary immunodeficiency seen in CKD exhibits a relatively complex phenotype and does not simply reflect global immune failure. Indeed, humoral immunity to historical and persistent antigens is well maintained and may suggest that the cellular immune response to these antigens is also preserved. As such these findings suggest that future efforts to improve immune function in patients with CKD should focus on seeking to improve the adaptive immune response to new antigenic challenges, such as those observed during seasonal influenza infection or administration of novel vaccines. These efforts will be supported as a more complete understanding of the full phenotype and cause(s) of the secondary immunodeficiency seen in less severe stages of CKD is developed.

## Materials and methods

### Subject selection

#### Cohort 1

A random sample of 60 subjects was selected from all study patients that had completed 18 month follow-up for the Renal Impairment In Secondary Care (RIISC) study [50] in August 2013. RIISC is a prospective, observational cohort study of adult patients managed in secondary care with advanced and/or progressive CKD, aiming to identify determinants of renal disease progression through detailed bio-clinical phenotyping. Patients with immune-mediated renal disease and those undergoing immunosuppression or RRT were not eligible for recruitment to RIISC.

All patients with a history of malignancy (except non-melanoma skin cancer) were then excluded from the sample, as was an individual with incomplete baseline clinical data. Clinical and laboratory data (including medical and drug history, baseline renal biochemistry and full blood count), together with baseline serum samples were sourced for the final sample of 43 RIISC patients. Blood samples from RIISC were collected as per previously published protocols. [50]

Clinical and laboratory data, together with serum samples from healthy control subjects recruited to a study investigating physical fitness in subjects with ANCA-associated vasculitis [51] were used as the control group and age-matched to our CKD sample cohort (n = 39). Individuals with any major organ disease were not eligible for recruitment as controls in the vasculitis study. Serum samples were from blood drawn in the morning immediately prior to exercise testing as part of the vasculitis study. Blood samples were allowed to clot for 30–60 minutes and then spun at 5000rpm for 10 minutes at 4°C prior to aliquoting and storage at -80°C.

#### Cohort 2

A separate cohort of older patients (over 65 years) with CKD and age-matched controls was used to verify serological trends seen in cohort 1—baseline serum samples were tested from individuals taking part in the SONIC study (investigating the immune System in chrONIC kidney disease, clinicaltrials.gov ID: NCT02535052) between 2015 and 2016 –a prospective observational cohort study utilising clinically recommended seasonal vaccinations to evaluate host immune function.

Use of data and samples for this study falls under Ethical approvals granted to the above-named studies (RIISC approved by the South Birmingham Local Research Ethics committee–reference 10/H1207/6; vasculitis study approved by the Birmingham, East, North, and Solihull Research Ethics Committee–reference 09/H1206/113; SONIC approved by the Edgbaston Research Ethics Committee—reference 15/WM/0057) and written informed consent was obtained from all patients in accordance with the Declaration of Helsinki.

### Multiplexed functional antibody assay

This assay was performed as previously described [24] using a mix of carboxylated fluorescent microbeads specific for distinct bead regions on a Luminex instrument (BioRad, Hercules, USA) each conjugated to one of two antigens (DT and TT, both at 50μg/ml). The intra- and inter-assay variability for this IgG assay are 8% and 31% respectively. [24]

### Cytomegalovirus ELISA

Serum anti-CMV IgG titres were determined using a semi-quantitative in-house ELISA as previously described. [52] Briefly, diluted cell lysate purified from CMV-infected fibroblast cultures and uninfected cells were used to coat a 96 well plate. Samples were added in a 1∶600 dilution for cohort 1 sera and in a 1:1500 dilution for cohort 2 sera (due to a change in the in-house standard operating procedure over time) together with standards for a calibration curve (pooled plasma from three healthy CMV positive donors) and incubated for 30 minutes at room temperature. Secondary antibody (anti-human IgG-horseradish peroxidase—Southern Biotech, Birmingham, AL, USA) was added after washing the plate with phosphate buffered saline (PBS)/0.05% Tween20, and incubated for a further 30 minutes at room temperature (RT). The plate was developed with tetramethylbenzidine solution and read using a microplate reader at absorbance 450 nm. Optical density attributable to CMV IgG only was calculated by subtracting control lysate well values from the CMV lysate wells. A cut off of 10 arbitrary units was used to determine CMV IgG seropositivity.

### Lipopolysaccharide (LPS) ELISA

An in-house ELISA was used to semi-quantitatively determine the presence of anti-LPS IgG in serum, relative to a single healthy donor positive control used as an internal standard. Briefly, a 96-well Nunc Maxisorp plate was coated overnight at 4°C with 5μg/ml LPS from SEn (Enzo Life Sciences, Farmingdale, NY, USA) in a carbonate/bicarbonate coating buffer (Sigma Aldrich, St. Louis, MO, USA). The plate was washed 3 times with PBS and blocked with PBS/1% bovine serum albumin (BSA) for 1 hour at RT, agitated on a plate rocker. After a further 3 washes with PBS, test serum added in single replicates of six 4-fold dilutions– 1:20, 1:80, 1:320, 1:1280, 1:5120, 1:20480, diluted in PBS/1%BSA/0.05% Tween 20. Each plate included a negative control (dilution buffer) and a positive biological control (healthy control serum used as internal standard). The plate was incubated at RT for 1 hour on a plate shaker and then washed four times with PBS/0.05% Tween 20. Alkaline phosphatase conjugated secondary antibody (goat anti-human IgG -Sigma Aldrich, St. Louis, MO, USA) was added at 1:6000 and the plate incubated for a further 1 hour at RT on a plate shaker. P-nitrophenylphosphate (Sigma Aldrich, St. Louis, MO, USA) was then added and the plate incubated for 1 hour on a plate shaker at RT. The reaction was stopped using 3M sodium hydroxide and the plate read at an absorbance of 405nm.

LPS titre from this assay is reported relative to the positive control serum and drawn from a single experiment. Sera from individuals with high/low relative LPS titres from this assay were run in 2 independent experiments to verify the trend. The intra- and inter-assay coefficients of variation for this assay were 2.11% and 8.11% respectively.

### *Salmonella* Enteritidis serum bactericidal killing assay

This was performed as previously described [45] using SEn strain D24954. Briefly, log phase bacterial culture was prepared to a concentration of approximately 10^7^ colony forming units/ml and 5μl was added to 45μl neat sample serum in a 96 well plate, which was incubated on a plate rocker at 37°C, 5% CO_2_. Aliquots of 10μl were removed from each sample well at 45, 90 and 180 minutes and diluted in PBS into four serial 10-fold dilutions. Each dilution was pipetted in triplicate onto sterile Lysogeny agar plates, allowed to dry and incubated overnight at 37°C, 5% CO_2_. Colonies were counted the following day and the starting concentration of bacteria was determined by Miles & Misra serial dilution.

An increase in bacterial count from baseline denoted bacterial growth, whereas a decrease denoted bacterial killing. Each SBA contained one positive (healthy control serum with known high bacterial killing) and one negative control (antibody depleted healthy control serum–prepared as described previously). [27] Normal serum killing was defined as a greater than 1.5 log10 reduction in bacterial count from baseline at 180 minutes.

### Statistical analysis

Statistical analysis of data was performed using Prism 5 (GraphPad, San Diego, CA, USA) and SPSS 22 (IBM, New York, NY, USA). For continuous data, Normality has not been assumed and results are presented as medians with inter-quartile range (IQR) unless otherwise stated. The non-parametric Mann-Whitney test has been used to compare continuous numerical data and Fisher’s exact test to compare categorical data. Correlation analysis was performed using the non-parametric Spearman’s rank test. Multivariate linear regression was used to model predictors of CMV IgG titres in seropositive individuals and a repeated measures ANOVA was used to evaluate bacterial killing in the SBA.
